# Business Intelligence and the export competitiveness of lambayecan SMEs in the north of Peru

**DOI:** 10.12688/f1000research.167062.1

**Published:** 2026-03-06

**Authors:** Rogger Orlando Morán-Santamaría, Yefferson Llonto-Caicedo, Christel Lucero Choque-Yarasca, Nilda Rosa Barrutia-Montoya, Leydi Jhohania Bardales-Torres, Lizbeth Ivonne Gil-Contreras, Sheyla Johana Chavesta-Paico, Rubén Iván Marchena-Chanduvi, Diana Marilú Sánchez-Alamo, Isaí Geremias Ruiz-Díaz, Nikolays Pedro Lizana-Guevara

**Affiliations:** 1 Programa de Doctorado en Administración de la Escuela de Pos-grado, Universidad Nacional Pedro Ruiz Gallo, Lambayeque, Lambayeque, Peru; 2 Facultad de Ciencias Económicas, Administrativas y Contables, Universidad Nacional Pedro Ruiz Gallo, Lambayeque, Lambayeque, Peru; 3 Facultad de Administración y Negocios, Universidad Tecnologica del Peru, Lima, Lima, Peru; 4 Escuela Profesional de Ingeniería Agroindustrial, Facultad de Ciencia Agrarias, Universidad Nacional Autonoma de Chota, Chota, Cajamarca, Peru; 5 Escuela Profesional de Administración de Negocios Globales, Universidad Nacional Intercultural Fabiola Salazar Leguia de Bagua, Amazonas, Peru; 6 Escuela Profesional de Ingeniería en Agronegocios, Universidad Nacional de Cajamarca, Cajamarca, Cajamarca, Peru

**Keywords:** Business Intelligence, Competitiveness, Exports, Lambayeque, Lambayeque, Logit model

## Abstract

**Background:**

International expansion has been identified as a pivotal strategy for SMEs seeking to achieve internationalisation and growth within the global market. However, as evidenced by extant literature, SMEs encounter numerous obstacles that impede their ability to access foreign markets, including a dearth of resources and experience, fierce competition from large and sustainable global companies. Therefore, the study seeks to identify the Business Intelligence factors that affect the export competitiveness of SMEs in a region of northern Peru.

**Methods:**

The research had a quantitative, non-experimental and explanatory approach; being basic research focusing on the generation of theoretical knowledge about the factors that affect competitiveness through business intelligence strategies, for which 51 SMEs, economic units that currently maintain trade relations with international markets, were studied and the Logit model was used to identify those factors.

**Results:**

The findings indicate that the binary logistic regression model demonstrates the significance of the market research and business management dimensions. The assertiveness matrix demonstrates a higher capacity to predict the 'No' response (76.5%) in comparison to the 'Yes' response (23.5%). It is noteworthy that the relative probability of enhancing competitiveness is estimated at 0.98 times when market identification, target market segment analysis and market research development are requested, as opposed to 0.95 times when external advice or assistance is obtained to formulate an alternative value proposition for the exportable product.

**Conclusions:**

The market research and business management dimensions of Business Intelligence have a significant impact on the export competitiveness of SMEs in northern Peru, with market research being the factor with the greatest influence on the export competitiveness of SMEs. This finding highlights the importance of collecting and analysing accurate information on international markets in order to identify opportunities, reduce uncertainties and adapt products to the demands of global customers.

## Introduction

In the contemporary globalised context, the imperative for competitiveness necessitates that firms embrace innovation to navigate the increasingly stringent international markets. ^1^ International expansion has been identified as a pivotal strategy for MSEs seeking to achieve internationalisation and growth in the global market. However, as indicated by extant literature, SMEs encounter challenges such as a paucity of resources and experience, leading to difficulties in accessing foreign markets. Furthermore, SMEs face competition from large, sustainable global companies, as well as logistical difficulties and regulatory variations across different markets. Consequently, enterprises are compelled to respond to these challenges and are required to be empowered by innovation, a concept that has been propelled in the last two decades by the advancement of information and communication technologies (ICT), resulting in a transformation in business. ^2– 4^

These technologies have had a profound impact on various aspects of society, including everyday life, ^5^ interpersonal interactions, and business practices. They have facilitated the development of products, processes, and tools that have in turn enabled the adoption of digital business models in companies of all sizes. ^6– 8^ As posited by the Ref. Bagheri et al. ^9^ the conception of innovation extends beyond the mere integration of technological elements. Rather, it encompasses a more profound and comprehensive paradigm, encompassing the identification of novel market requirements and the discernment of products with the potential for exportation that meet stringent quality standards. Consequently, it is imperative that companies receive information at the optimal time and make it accessible, enabling them to make optimal decisions and obtain competitive advantages, thereby positioning themselves in the various international markets. ^10, 11^

It is an irrefutable fact that in the aftermath of the global pandemic, companies in developing countries have adopted Business Intelligence (BI) as a dynamic strategy to keep abreast of changes in every market environment, thereby maintaining competitiveness in the face of the challenges posed by globalisation and increasing international trade. ^12, 13^ In this context, Business Intelligence (BI) encompasses sources, databases, analytical tools and applications that seek to provide and integrate data in real time, providing valuable information for managers to convert them into decisions that are useful in business actions. ^14^

In recent years, Business Intelligence (BI) has experienced a favourable and significant scenario in terms of competitive advantage through an integrated four-stage model, encompassing data, information, knowledge and decisions. This model enables organisations to investigate and extract data and information, thereby detecting business opportunities and minimising external threats in the face of uncertainties in different economies globally. Consequently, this improves knowledge of foreign markets and export performance. ^15– 18^ International cooperation among BI companies becomes imperative for SMEs to enhance their development, as many of these companies are deficient in resources for the development of intelligent systems. Collaboration with these multinational BI companies will mitigate uncertainties concerning export behaviour, access barriers, market prices, market analysis, among others, thereby promoting international sustainable growth. ^19, 20^

A significant issue in the implementation of BI on a global scale is the disparity in technological capabilities between companies in different countries. While large corporations in developed countries are able to invest in advanced BI solutions, many companies in developing countries encounter challenges in accessing these technologies due to financial constraints and a lack of infrastructure. ^21^

As previously discussed, the discrepancy in the capacity to adopt and utilise sophisticated business intelligence tools engenders an inequitable competitive environment. Large corporations, which invest approximately 35% more in information technology than small and medium-sized enterprises, as reported by Gartner, have access to superior tools and analytics, thereby strengthening their position in the global market and creating significant barriers to entry for small businesses. ^22, 23^ This predicament is further compounded by the fact that many small businesses find themselves ill-equipped to compete effectively on the global stage, where advanced analytical skills have become increasingly indispensable. These challenges underscore the necessity for a more integrated and cooperative approach at the international level to enhance the interoperability of business intelligence systems and the quality of available data. ^24^ The implementation of global standards for data exchange, and the investment in technologies that facilitate better data integration, would be essential steps to mitigate these issues and improve export competitiveness through the use of business intelligence.

Exporting is an essential factor for business survival and growth; however, in the face of accelerated competitive dynamics, internationaLilisation has become a crucial strategy for small firms. ^25, 26^ It is estimated that Latin American SMEs account for 96.6% of all firms, contributing 28% of GDP and generating a significant impact on industry and employment. However, their export share is 13% lower than in other countries such as Korea (19%), Thailand (47%) and Malaysia (55%). ^27^ Empirical evidence from scenarios in Latin American countries within the Pacific Alliance highlights the pivotal role played by Colombian small firms in the economy, accounting for 37.2% of the country's total exports. However, these small firms encounter constraints due to limited resources, risk aversion, and a deficiency in understanding the opportunities that facilitate their operations in accessing foreign markets. ^28^

In the Peruvian context, enterprises encounter a plethora of obstacles when attempting to effectively implement business intelligence within their export strategies. A significant challenge pertains to the inadequate technological infrastructure, which hinders the capacity to collect and process data on a substantial scale. This limitation also obstructs the development of sophisticated analytics essential for competitiveness in global markets. Additionally, Peru occupies a median position in terms of ICT adoption, indicative of constraints in terms of access to and utilisation of advanced technologies. ^29^

A further salient issue in Peru pertains to the absence of a comprehensive data culture among Peruvian firms, particularly within the domain of small and medium-sized enterprises, which collectively constitute the preponderance of the nation's business landscape. The training in data analysis and the utilisation of business intelligence tools is still in its infancy. This has resulted in a limited adoption of these technologies within the export sector. Furthermore, it has been determined that merely approximately 20% of SMEs employ some form of business intelligence in their operations, a figure which considerably restricts their capacity to identify market opportunities and optimise their export strategies. ^30, 31^

The survival rate of Lambayeque agro-exporting enterprises is low, with 50% ceasing to operate after one year, 30% only surviving to the second year, while a mere 22% survive to the third year. During the ten-year period, a mere 4% of these enterprises were able to maintain continuous operation. ^32^ The absence of sustainability and business competitiveness in the field of exporting SMEs in Lambayeque can be attributed to three key factors. Firstly, there is a dearth of awareness regarding international market trends. Secondly, there is an absence of trade support policies designed to attract customers. Thirdly, there is a paucity of tools to facilitate foreign trade. The absence of a business intelligence system has been demonstrated to limit competitive export management, generating obstacles in the export process. ^33, 34^ In this context, the research problem was: What are the Business Intelligence factors that affect the export competitiveness of SMEs in a region of northern Peru?

Against this background, the main objective of this research was to determine the Business Intelligence factors that have influenced the export competitiveness of SMEs in a region of northern Peru, focusing particularly on describing the probabilistic incidence of Business Intelligence strategies being applied by SMEs in the Lambayeque region in the development of exports to the world, because despite having a great diversification of exportable supply, there are few scientific studies that allow us to verify whether the increase in exports is a result of the application of Business Intelligence.

## Theoretical and empirical review of related literature

**Figure 1.**
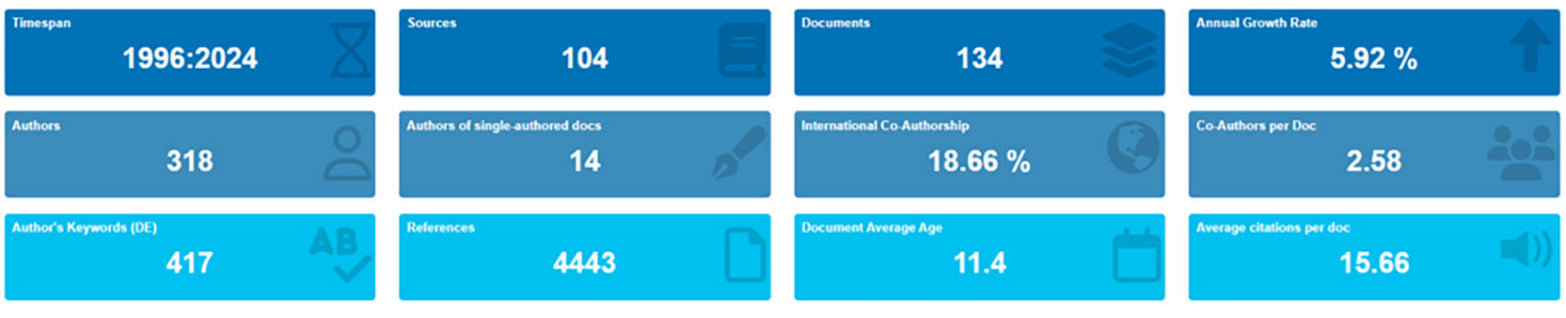
Global bibliometric metrics of scientific production in trade intelligence and exports according to Scopus (1996-2024).

The analysis of the literature review of the research was focused through the Scopus database, where related terms were combined by creating a search equation: ( TITLE-ABS-KEY (‘business intelligence’ OR ‘market intelligence’ OR ‘business intelligence’)) AND (TITLE-ABS-KEY (export* OR ‘export’ OR ‘exports’)). The empirical review shows that from 1996 to 2024 only 134 scientific articles published in 104 scientific journals indexed in Scopus were identified, reaching an average annual variation of scientific production of 5.92% and with a degree of international co-authorship of 18.66%. These data denote that there is a moderate amount that address the subject of research, but little limited, compared to the scientific maturity such as artificial intelligence that exceeds 40% and 50% compared to the modest 18.66% found, so it is expected to grow scientific collaboration on a regular basis by affiliated authors from different countries ( Figure 1).

In accordance with the metrics identified in the extant literature, the theoretical contribution of Business Intelligence is posited as a set of methodologies, processes, architectures and technologies that transform raw data into meaningful and useful information that supports strategic business decisions. ^35^ This discipline encompasses a range of activities, including data acquisition, analysis, and presentation of analytical information, with the overarching objective being the enhancement of decision-making processes within organisations. ^36^ Business Intelligence (BI) technologies encompass a variety of software solutions, including decision support systems, executive information systems, query and reporting tools, online analytical processing tools, and data mining software. The importance of business intelligence in the business world lies in its ability to enable organisations to make effective use of their accumulations of data. By transforming data into meaningful information, companies can identify market opportunities, optimise existing processes, increase operational efficiency and maintain sustainable competitive advantages. ^37^

Furthermore, Business Intelligence (BI) plays a pivotal role in enhancing the financial performance of any organisation. Indeed, by leveraging precise, real-time analytics, executives are strategically positioned to make decisions that exert a positive influence on the bottom line. For instance, the implementation of BI analytics enables a company to identify and reduce superfluous expenditures, substantially optimise its supply chain, and consequently enhance customer satisfaction. It is evident that these factors contribute to a substantial increase in revenue. ^38^

Business Intelligence (BI) is a multifaceted concept that encompasses a number of crucial strategies for effective implementation. ^39^ The initial strategy emphasises business intelligence, defined as the aggregation, examination, and administration of an organisation's internal data. This encompasses a wide range of information, including but not limited to, production yields, operational efficiencies, and financial performance. This particular dimension of BI places emphasis on the optimisation of internal processes, with the ultimate goals of enhancing quality and productivity. ^40^ The effective employment of business intelligence by exporting firms has been demonstrated to facilitate enhanced strategic and operational planning, optimisation of resource utilisation and cost reduction, thereby directly contributing to enhanced competitiveness in international markets. ^41^ The significance of business information lies in its capacity to furnish a robust and impartial foundation for decision-making. In the agro-products sector, characterised by extensive and intricate operations, the effective management of information is paramount to ensure sustained competitiveness. ^42^

Another BI strategy is market intelligence, which is defined as the systematic process of collecting, analysing and interpreting information about the market environment, including competitors, customers, economic factors and trends. ^43^ The significance of this strategy lies in its emphasis on external factors, providing crucial data for comprehending and anticipating market dynamics. In the context of agricultural products, this includes understanding consumer preferences, import/export regulations, and competitors' strategies. ^44^

Conversely, the third BI strategy of business management entails the planning, implementation and control of a company's sales and distribution activities. The focus of this dimension is on the optimisation of revenues through the effective marketing of products and services. The scope of the dimension extends from the definition of pricing strategies to the management of customer relationships and the optimisation of the distribution chain. ^45^ Consequently, this strategy is imperative to guarantee that products not only access international markets, but also satisfy the globally stipulated quality and sustainability standards, thereby sustaining customer loyalty and enhancing brand image in foreign markets. ^46^

The theoretical basis of the research can be strengthened and expanded by integrating the findings of recent studies. Some research based on findings related to the impact of Business Intelligence on export competitiveness efficiency, authors such as ^47^ highlighted that to achieve a sustainable competitive advantage, emerging markets must implement strategies such as IT systems, public policies and business intelligence (BI), which are crucial for the short- and long-term survival of agricultural businesses, fostering business opportunities in competitive international markets. Furthermore, the study conducted by Eidizadeh et al. ^15^ shows a conceptual bridge highlighting that Business Intelligence has a very significant impact on knowledge exchange and innovation, giving exporting companies a competitive advantage. For example, the study conducted by Anjaningrum et al. ^48^ found that small businesses in developing countries, despite limited resources, apply business intelligence, albeit in a simple way, managing to apply innovation and achieving high export performance compared to large, highly competitive companies. Heriqbaldi et al. ^49^ highlighted that the effect of export promotion programmes (EPPs) is relatively significant in small companies and those with more export experience. They also confirm that EPPs have the most significant impact on firms' resources and capabilities, and that assistance programmes aimed at improving organisational capabilities are needed to enhance marketing strategies. Although innovative capabilities and business intelligence offer great potential to support export performance, EPP-type assistance programmes have not been adequately developed. Aunque las capacidades innovadoras y la inteligencia empresarial ofrecen un gran potencial para apoyar el desempeño exportador, los programas de asistencia tipo PPE no han sido adecuadamente desarrollados. Antoniadis et al. ^50^ add that the capabilities that a company acquires through business intelligence capabilities are indeed considered competitive, as they highlighted that data provided by foreign trade databases are very important for the preparation of export and import trade reports. However, factors such as culture, organisational leadership, learning and quality are significantly affecting business performance, leading to missed opportunities in the face of the different needs created by high global demand. These findings integrate BI theory, highlighting, as Cheng et al. ^51^ point out in their findings, that business intelligence significantly influences a changing world during internationalisation.

In consideration of the findings, it is evident that the international competitiveness of small businesses is contingent on their approach to competitive business intelligence. This approach encompasses supplementary imperative actions that are not solely defensive in nature but also have the potential to be business-oriented. In particular, the integration of business competitive intelligence as an additional factor affecting the internationalisation process has been identified. ^52^ This is in addition to the classic factors defended by global birth theory and network theory. The competitive position of small businesses is contingent on the approach to business competitive intelligence, as it encompasses additional essential actions that extend beyond a mere defensive attitude, encompassing a business orientation. ^53, 54^ Consequently, these findings align with the notion that commercial intelligence encompasses the exploration of new international markets, thereby reducing small businesses' reliance on domestic markets. ^55^

## Methodology

### Type, design and scope

The present research adopts a quantitative, non-experimental, explanatory approach. This approach is employed to identify and explain the causes of the phenomenon, to determine the conditions under which the phenomenon occurs, and to establish its relationship to the variables and dimensions. The objective is to analyse and interpret quantifiable data in order to determine the Business Intelligence factors that have influenced the export competitiveness of SMEs in a region of northern Peru, without altering the behaviour of the variables. ^56– 58^ The research adopted a basic type of research, focusing on the generation of theoretical knowledge about the factors that affect competitiveness through business intelligence strategies related to business information, market intelligence and business management. ^59^

### Population and sample

**Figure 2.**
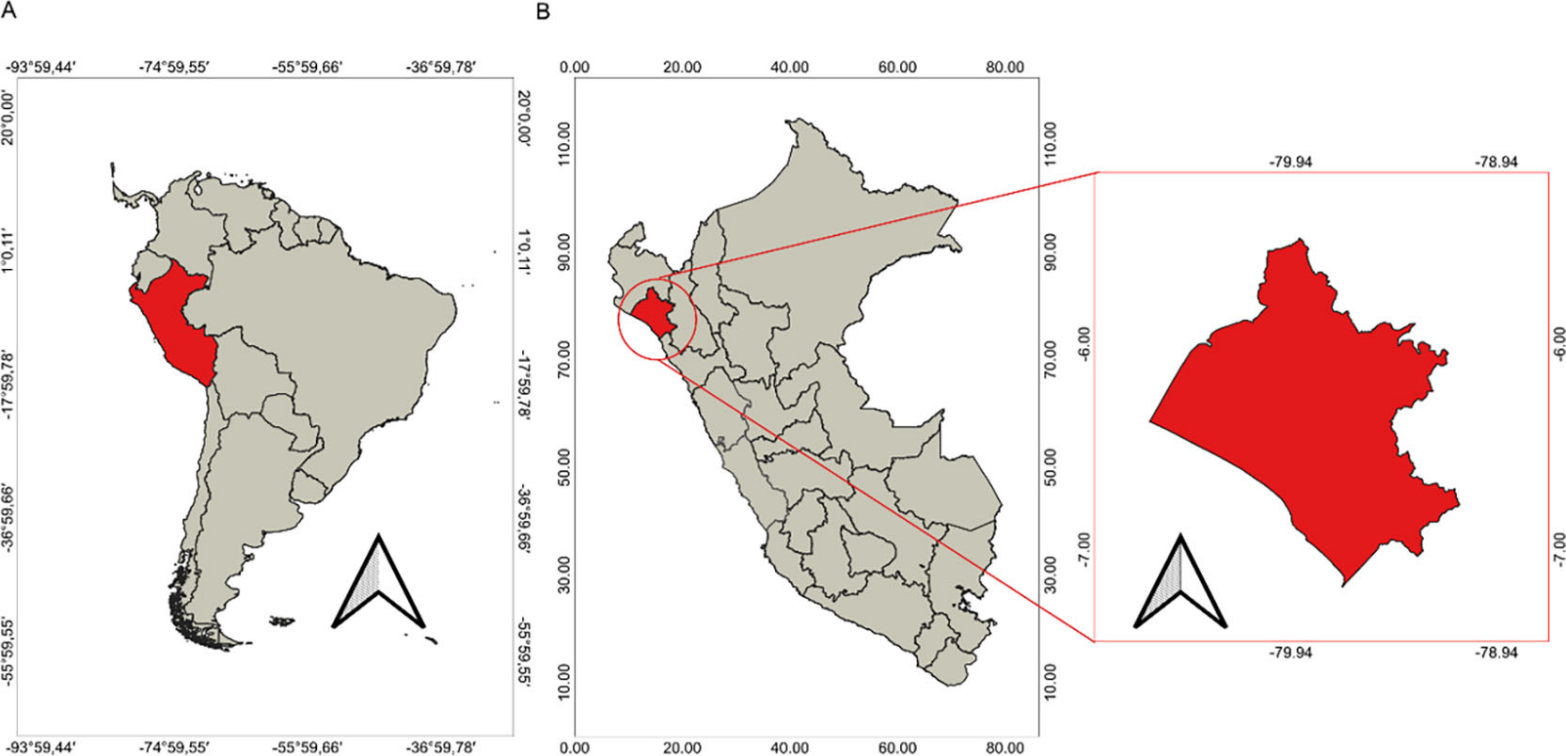
Geographical location of Lambayeque. A: Geographical location of Peru. B: Geographical location of Lambayeque.

This study was conducted in the Lambayeque region, which is located in northern Peru. ^60^ The region under discussion here covers an area of 14,000 km ^2^ and is estimated to have a population of 1,112,868 inhabitants. Administratively, the Lambayeque region is comprised of three provinces: Chiclayo, Lambayeque, and Ferreñafe; and 38 districts ( Figure 2). ^61^

The population of SMEs in the Lambayeque region of Peru was determined to be 106 exporting entities, ^62^ with 51 of these SMEs currently maintaining commercial relations with international markets, including the United States, the Netherlands, the United Kingdom, Spain, China, Ecuador, Hong Kong, Germany, Chile, Belgium, Japan, South Korea, Colombia, and others.

Table 1 presents the stratified distribution of the selected sample by sector. The sample was clustered, with the exporting SMEs being randomly selected from the total population of MSMEs located in the Lambayeque region (an agro-export hub) of Peru.

**Table 1.** Sample characteristics.

Sectors	# Companies	Percentage	Sample
Agriculture and Livestock	65	61%	31
Coffee	7	7%	3
Molasses	6	6%	3
Textile	6	6%	3
Fishing	5	5%	3
Mechanical metal	7	7%	3
Non-metallic mining	5	5%	3
Chemist	3	3%	1
Miscellaneous (inc. Jewellery)	2	2%	1
TOTAL	106	100%	51

Note: Number of exporting SMEs by sector in the Lambayeque region.

The procedure used to obtain the results began with the development and validation of the questionnaire, establishing variables, dimensions, and indicators. The surveys were structured and directed at the managing director and/or export operations manager of exporting SMEs in the region. Data was collected using various methods, including personal surveys, email correspondence, and Google forms. The reliability of the instrument was determined using Cronbach's alpha coefficient, in which the questionnaire obtained a result of 0.66, equivalent to 0.7, which is considered reliable.

Ethical approval and informed consent in this study was a minimal-risk, non-interventional investigation based on anonymous surveys in adults, without clinical procedures, experimental manipulation, or collection of sensitive or directly identifiable data. The work was developed as voluntary academic research within the Graduate School of Pedro Ruiz Gallo National University (UNPRG) and was not submitted as a thesis-/degree-related protocol for formal ethics committee review; therefore, no ethics committee/IRB approval, waiver, or reference number is available. Institutional ethics guidance is publicly available (UNPRG Code of Ethics and Scientific Integrity for Research—University Council Resolution No. 623-2021-CU: https://www.unprg.edu.pe/univ/portal/documentos_s/RESOLUCION%20N%C2%B0%20623-2021-CU.pdf; Regulation of the Research Ethics Committee—University Council Resolution No. 624-2021-CU: https://www.unprg.edu.pe/univ/portal/documentos_s/RESOLUCION%20N%C2%B0%20624-2021-CU.pdf; normative index page: https://www.unprg.edu.pe/vri_normativainterna.php). The study was conducted in accordance with the principles of the Declaration of Helsinki. Informed consent was obtained prior to participation: for online administration, electronic informed consent (e-consent) was provided on the first page of the Google Form; for in-person administration, participants reviewed a written information sheet and provided written consent. Participation was voluntary, responses were anonymised through coding, and participants could withdraw at any time without consequences.

### Data analysis

The data were analysed using the free software IBM SPSS Statistics 27. ^63^ An academic licence is available for the use of Stata 16 software. The licence details are as follows: Serial number: 501809389697. The software can be downloaded from the following link: https://download.stata.com/

In order to facilitate in-depth data analysis, each response provided for the variables commercial intelligence and export competitiveness was analysed, thereby generating knowledge and understanding of the research. ^64, 65^ The Logit model was utilised to ascertain the impact of BI factors on competitiveness, in accordance with the following structural equation: where Business competitiveness is a dummy variable (dependent or endogenous) that assumes the value 1 when the company is competitive and 0 when it is not. ^66– 68^

The data were analysed using the free software IBM SPSS Statistics 27. ^63^ In order to facilitate in-depth data analysis, each response provided for the variables commercial intelligence and export competitiveness was analysed, thereby generating knowledge and understanding of the research. ^64, 65^ The Logit model was utilised to ascertain the impact of BI factors on competitiveness, in accordance with the following structural equation: where Business competitiveness is a dummy variable (dependent or endogenous) that assumes the value 1 when the company is competitive and 0 when it is not. ^66– 68^

Competitiveness=β0+β1information+β2market research+β3commercial management

According to the natural Logit model, according to Gómez Mejía ^69^ in an econometric model that estimates probability and predicts any event, optimally interpreting the coefficients. This test is represented by the following formula $\mathrm{Pi}=E\;\left(Y=k\right)=\beta_{1}+\beta_{2}\mathrm{Xi}$ . We can interpret that Pi represents the probability that an event, Y, will reach a value K, ranging between zero and one. Xi is the explanatory variable, while β1 and β2 are the estimated coefficients of the intercept and the slope.

## Results

### Resultados descriptivos

Table 2 provides a detailed description of the utilisation of business intelligence (BI) in Peruvian exporting SMEs. It meticulously delineates the distribution of probability levels designated as ‘Yes, Propensity’ and ‘No Propensity’ across the three dimensions. Firstly, within the Commercial Information Dimension, recent studies have emphasised that it encompasses Business Intelligence (BI) applications and tools. This refers to a company's ability to explore data mining, which allows it to collect and analyse structured data based on transactions. This process improves and uses this information to make strategic decisions in export business operations. ^70, 71^ The findings highlighted that the majority of companies (64.7%) acknowledged that their performance in this dimension was not satisfactory, suggesting a limited capacity to utilise business intelligence in their export trade operations. However, only 35.3% of companies are at a level where they apply this commercial information to their export propensity, suggesting a significant gap in interest among these companies in terms of data collection and analysis.

**Table 2.** Business intelligence (BI) variable.

Business information at binary dimension		International market research at binary dimension		Commercial management at binary dimension	
No Propensity % of N of row	Yes, Propensity % of N of row	No Propensity % of N of row	Yes, Propensity % of N of row	No Propensity % of N of row	Yes, Propensity % of N of row
64,7%	35,3%	45,1%	54,9%	68,6%	31,4%

Note. Obtained from the processing of the surveys.

Secondly, within the context of the International Market Research Dimension, recent studies have elucidated the concept of a company's capacity to undertake market research in diverse countries and regions. This capacity entails the discernment of risks and opportunities, enabling the company to respond effectively to international competition. The possession of this capacity fosters business efficacy by equipping the company with a comprehensive understanding of market size, market trends, market access, market analysis, and the formulation of strategies to address competitive conditions. Consequently, this fosters the achievement of success in the target market. ^72, 73^ The findings indicate that the majority of companies (54.9%) demonstrate a favourable standard level in this dimension. However, a discrepancy of 45.1% has been identified between companies encountering challenges in conducting international market research. This may be attributable to a paucity of resources, experience or knowledge of foreign markets.

Finally, in the Commercial Management Dimension, recent studies describe this as a dimension that refers to a company's ability to manage its commercial activities effectively. Through BI evaluation and planning, companies improve their export performance. ^74, 75^ In this case, the results achieved indicate that the majority of companies (68.6%) are performing below average in this dimension, demonstrating substandard commercial management performance. However, a small percentage of companies (31.4%) have achieved a level of good practice, suggesting that the implementation of business intelligence strategies may facilitate the establishment of adequate capacity to manage internationalisation commercial activities.

The findings indicate that the majority of companies possess a moderate capacity to utilise international market intelligence. However, they also encounter challenges in analysing international commercial information and commercial management. This finding indicates that companies may benefit from enhancing their capabilities in these final two domains to capitalise on foreign market opportunities.

As illustrated in Table 3, the study examines the contribution of information services, market research and commercial management to business competitiveness. These factors are then classified into two probabilities. The categories employed in this study are 'Yes, Propensity' and 'No Propensity'.

**Table 3.** Business competitiveness.

Contribution level of the business information service in business competitiveness		Level of contribution of the international market research service to business competitiveness		Contribution level of the commercial management service in business competitiveness	
No % of N of row	Yes % of N of row	No % of N of row	Yes % of N of row	No % of N of row	Yes % of N of row
76,5%	23,5%	17,6%	82,4%	72,5%	27,5%

Note. Obtained from the processing of the surveys.

The findings of the study indicated that the majority of companies (76.5%) do not perceive information services as contributing to their competitiveness. A mere 23.5% of companies assessed them as being of average quality. This finding suggests that many companies are not leveraging the full potential of BI commercial information services to enhance their competitiveness, which is not a viable long-term strategy in foreign markets.

Market research: The majority of companies (82.4%) posit that market research is instrumental in enhancing competitiveness. The findings suggest that market research is being utilised effectively in numerous organisations to achieve a competitive advantage over national and global rivals.

The management of commercial entities. As was the case in the two preceding instances, the majority of companies (72.5%) have expressed the opinion that commercial management does not contribute to competitiveness. This finding indicates that the strategic utilisation of commercial management in numerous corporations warrants enhancement to facilitate successful entry into foreign markets.

It is evident that the majority of companies are capitalising on the potential of information services and commercial management to enhance their competitiveness. However, micro-enterprises are not leveraging market research to its full potential, which represents an opportunity for improvement for many companies, as it could enable them to achieve a better position in international markets.

*Results with the logistic regression model*

The survey items sought to identify the weight of business intelligence components in the current competitiveness of small exporting firms.

In order to analyse the probabilistic incidence of business intelligence on the competitiveness of Peruvian exporting SMEs, a binary logistic regression model is used, expressed in the following formula.

$$P\left(\text{Competitiveness}\right)=\frac{1}{1+e^{\left(\beta 0+\beta 1\mathrm{IC}+\beta 2\mathrm{IM}+\beta 3\mathrm{GC}+\mathrm{\mu t}\right)}}$$

Where:

*Competitiveness:* represents the probability of the company being competitive, taking the value 1 if it is competitive and 2 if it is not competitive.

*Trade Intelligence Information (TI)*: represents the access to statistical information on the products that your company sells to the foreign market, taking the value 1 if you have access and 2 if you do not have access.

*Market research (MI)*: represents the opportunity to request market identification, target market segment analysis and market research development, taking the value 1 if requested and 2 if not requested.

*Commercial Management (CM)*: represents the receipt of external advice or accompaniment to develop a different value proposition for the exportable product, which allows it to compete in the international market, taking the value 1 if it is received and 2 if it is not received.

From the Wald forward stepwise methodology which involves recruiting the input based on the significance of the score statistic and contrasts the elimination based on the likelihood of the Wald statistic. Considering the binary logistic regression model which involves a cross-sectional analysis in the year 2024 it is obtained that the dimensions market research and business management are significant; while business intelligence information is not significant ( Table 4).

**Table 4.** Statistical significance and signs of the coefficients.

		B	Standard error	Wald	gl	Sig.	Exp(B)
Step 1 ^a^	Market research	2.420	0.695	12.120	1	0.000	0.089
	Constant	0.629	0.438	2.062	1	0.151	1.875
Step 2 ^b^	Market research	4.026	1.164	11.973	1	0.001	0.018
	Commercial Management	3.032	1.196	6.424	1	0.011	0.048
	Constant	2.453	1.041	5.552	1	0.018	11.625

^a^ Variables specified in step 1: International Market Research at Binary Dimension.

^b^ Variables specified in step 2: Commercial Management at Binary Dimension.

The goodness of fit of the model and the significance of the model is observed in the Nagelkerke R-Squared, where 52.1% of the variations in the probability of being competitive are explained by the dimensions market research and business management ( Table 5).

**Table 5.** Summary of the model.

Passage	Logarithm of the likelihood -2	Cox and Snell R-squared	R square of Nagelkerke
1	52,687 ^a^	0.250	0.341
2	42,662 ^b^	0.384	0.523

^a^ The estimation ended at iteration number 4 because the parameter estimates changed by less than 0.001.

^b^ The estimation ended at iteration number 6 because the parameter estimates changed by less than 0.001.

On the other hand, to analyse the significance of the model we observe that the Hosmer and Lemeshow test is greater than 0.05, so the null hypothesis is accepted ( Table 5).

Ho: The model is significant (Sig>0.05).

H1: The model is not significant (Sig<0.05).

In the Logit model, the R's of the total model are secondary, the values obtained were less than 0.5, evidencing that the Nagelkerke R-squared obtained a value greater than 0.5, explaining that the independent factors explain more than 50% of the probability of a company in the agro-export sector being competitive ( Table 6).

**Table 6.** Hosmer and Lemeshow test.

Passage	Chi-square	gl	Sig.
1	0.000	0	0
2	1.252	2	0.535

*Note.* Obtained from data processing in SPSS V.25.

The binary logistic regression model shows in the assertiveness matrix that it predicts better the No (96.9%) than the Yes (57.9%); being the model elaborated the one that has correctly classified 82.4% of the cases; that is to say it allows to evaluate the fit of the regression model; which shows a significant fit ( Table 7).

**Table 7.** League table. ^a^

Observed			Forecast		
			Business competitiveness at Binary Dimension		
			No Business competitiveness	Business competitiveness	Percentage correct
Step 1	Likelihood of being competitive	No	24	8	75.0
		Yes	4	15	78.9
	**Overall percentage**				**76.5**
Step 2	Likelihood of being competitive	No	31	1	96.9
		Yes	8	11	57.9
	**Overall percentage**				**82.4**

^a^ The cut-off value is 500.

The Odds ratios of the significant variables at 5% of the chosen model are obtained as a result ( Table 8):

• The relative likelihood of an increase in competitive status if it is requests for market identification, target market segment analysis and market research development is 0.98 times.
• The relative probability of increasing the condition of being competitive if external advice or accompaniment is received to develop a different value proposition for the exportable product is 0.95 times.

**Table 8.** Odds ratio of the binary logistic regression model.

		B	Standard error	Wald	gl	Sig.	Exp(B)	1- Exp (B)
Step 1 ^a^	Market research	-2.420	0.695	12.120	1	0.000	0.089	0.91
	Constant	0.629	0.438	2.062	1	0.151	1.875	
Step 2 ^b^	Market research	-4.026	1.164	11.973	1	0.001	0.018	0.98
	Commercial Management	-3.032	1.196	6.424	1	0.011	0.048	0.95
	Constant	2.453	1.041	5.552	1	0.018	11.625	

^a^ Variables specified in step 1: International Market Research at Binary Dimension.

^b^ Variables specified in step 2: Commercial Management at Binary Dimension.

**Figure 3.**
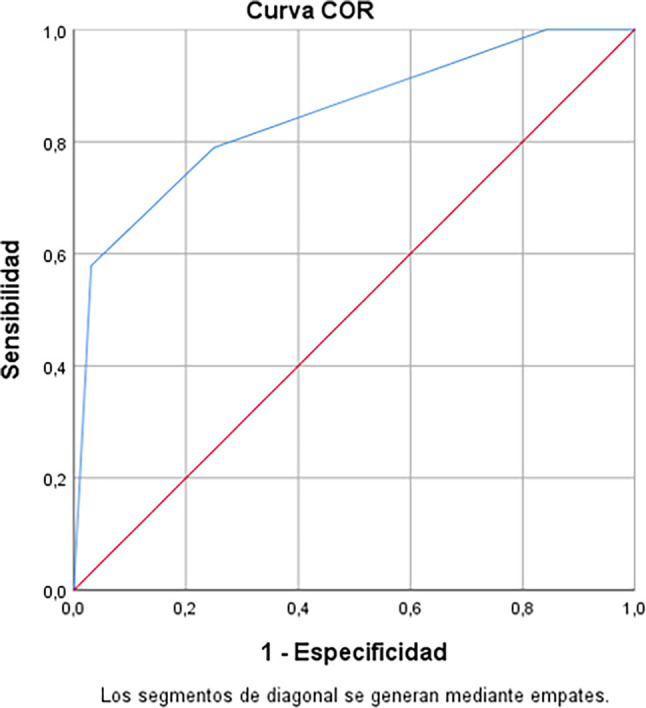
Sensitivity and specificity of the model.

The parametric COR curve shows the overall performance of a test (area under the curve), generating an overall performance of 84.6%. Thus the estimates centred on sensitivity, specificity and area under the curve, which for each point the best estimators are parametric; translating into the differential between being competitive and non-competitive, generates the ability to classify correctly is 84.6% ( Figure 3).

The blue curve rises above the red diagonal, showing the greater predictive capacity of the model (high sensitivity), also due to the identification of false positives on the specificity axis, so we proceeded to calculate the exact area under the curve, obtaining the following ( Table 9).

**Table 9.** Area under the curve: Predicted probability.

Area	Error ^a^	Asymptotic significance ^b^	95% asymptotic confidence interval	
			Lower limit	Upper limit
0.846	0.059	0.000	0.730	0.963

The test result variables: Predicted probability have, at a minimum, a tie between the actual positive status group and the actual negative status group. The statistics could be biased.

^a^ Under the non-parametric assumption.

^b^ Null hypothesis: true area = 0.5.

The area was 0.84, which accurately supports the predictive ability of the studied model, which can be useful to apply it to other industries or in other geographical areas and obtain an adequate discussion of results.

## Discussion of results

The results of the present research, which analyse the influence of Business Intelligence (BI) on the export competitiveness of MSEs from Lambayeque in northern Peru, converge with the findings of Eidizadeh et al., ^15^ who argue that BI generates a significant impact on knowledge sharing and innovation, which is crucial to obtain sustainable competitive advantages. In line with these results, the research confirms that the dimensions of market research and business management have a statistically significant impact on the export competitiveness of SMEs, as evidenced by the applied binary logistic regression model.

Consistent with Li and Lakzi, ^47^ our results underline that the implementation of business intelligence systems, together with appropriate public policies, enhances the efficiency of export competitiveness; specifically, the market research dimension shows a prominent effect on improving competitiveness, which is in line with these authors' assertion on the need to adopt a systematic approach to collect and analyse international market data.

On the other hand, the findings of this research diverge from the results obtained by Anjaningrum et al., ^48^ who argue that small enterprises in developing countries, despite limited resources, apply business intelligence in a simple but effective way to achieve high export performance; this study shows that SMEs in Lambayeque have significant deficiencies in the business information dimension, which limits their ability to compete efficiently in global markets.

The positive relationship between trade management and export competitiveness identified in this study is supported by the research of Heriqbaldi et al., ^49^ who argue that export promotion programmes (EPPs) have a significant impact on organisational capabilities and the implementation of marketing strategies. This study confirms that external advice and strategic accompaniment are determinant for the development of differentiated value propositions that improve the international competitiveness of SMEs.

In contrast, the findings of Antoniadis et al. ^50^ suggest that BI adoption is more effective in business environments with a mature and innovation-oriented organisational culture; however, our study reveals that MSEs in Lambayeque face structural and cultural barriers that hinder the full implementation of BI systems, which reduces their ability to take advantage of global market opportunities.

The logistic regression model applied confirms that market research has the greatest influence on the likelihood of an SMEs being competitive. This result is in line with the study by Bertrand et al., ^20^ who argue that access to accurate international market information enables small firms to overcome entry barriers and improve their competitive position in globalised environments.

The results also show that business management is a determining factor for export competitiveness, in line with the findings of Reyes González et al., ^76^ who highlight the importance of strategic planning and supply chain optimisation for success in international markets. This study highlights that specialised support allows SMEs in Lambayeque to adapt their products to the demands of the global market.

On the other hand, in contrast to the findings of Cheng et al., ^51^ who highlight the crucial role of BI in organisational agility to accelerate internationalisation, our study reveals that SMEs in Lambayeque have a low level of BI adoption, which slows down their responsiveness to the changing dynamics of the global market.

In terms of the business information dimension, the results reflect a low impact on export competitiveness, which contradicts Skyrius, ^39^ who argues that the effective integration of internal information is fundamental to optimise operations and strengthen competitiveness. This finding suggests the need to strengthen technological infrastructure and training in da ta analysis among MSEs in Lambayeque.

The disparity between the dimensions of BI is also reflected in the low use of advanced tools for data collection and analysis, which is consistent with Olszak (2022) ^77^ regarding the difficulties faced by firms in developing countries in implementing integrated BI solutions. This study confirms that the lack of financial and technological resources limits the ability of MSEs in Lambayeque to adopt these technologies.

In relation to business competitiveness, it is observed that SMEs that invest in external consultancy for business management are 0.95 times more likely to be competitive. This finding is consistent with the conclusions of Haddoud et al., ^19^ who emphasise that access to expertise is crucial for optimising export strategies and improving competitiveness.

At a practical level, our results suggest that public policies should focus on strengthening market intelligence training and facilitating access to BI technologies for SMEs. These findings are in line with the recommendations of Morán, ^34^ who stresses the need to implement specific support programmes to improve export competitiveness management.

The main contribution of this study lies in identifying the critical dimensions of Business Intelligence that affect the export competitiveness of SMEs in Lambayeque. The findings provide empirical evidence that supports the importance of market research and business management as determinants for improving export performance in globalised environments.

Limitations of the study include the use of a single database and the analysis of a sample limited to the Lambayeque region, which could restrict the generalisability of the results to other regions of Peru or to different productive sectors. Future research could broaden the geographical scope and consider other mediating variables that influence the relationship between BI and export competitiveness.

In terms of perspectives for future research, it is recommended to explore the impact of digital transformation on the adoption of BI, as well as to evaluate the effectiveness of market intelligence training programmes to improve the export competitiveness of SMEs. It would also be relevant to analyse the role of strategic alliances with technology companies in overcoming barriers to access to advanced BI tools.

## Conclusion

The present study has identified that the market research and business management dimensions of Business Intelligence have a significant impact on the export competitiveness of SMEs from Lambayeque in northern Peru. The findings, as deduced from the binary logistic regression model, corroborate the hypothesis that these two dimensions augment the probability of a company attaining competitiveness in international markets. This outcome serves to underscore the significance of implementing systematic market analysis and business process optimisation strategies for achieving success in the realm of exports.

The study concluded that market research is the most significant factor influencing the export competitiveness of SMEs. This finding emphasises the importance of collecting and analysing accurate information on international markets to identify opportunities, reduce uncertainties and adapt products to global customer demands. The absence of an organisational culture oriented towards market intelligence limits the capacity of these companies to face access barriers and improve their competitive position.

In a similar fashion, the dimension of commercial management exerts a substantial influence on export competitiveness, underscoring the importance of obtaining external counsel and strategic assistance for the development of diversified value propositions. It is evident that SMEs in Lambayeque that implement more advanced trade management strategies are better able to adapt their products to international standards, optimise their distribution chain and strengthen their relationships with customers in foreign markets.

Conversely, the business information dimension has been found to exert a negligible influence on export competitiveness. This finding indicates that, despite the fact that Lambayequean SMEs have access to internal information regarding their operations, they do not effectively utilise this data to inform their strategic decisions within the international market. This limitation underscores the necessity to enhance the technological infrastructure and training in data analysis.

The geographical focus of the study is limited to the Lambayeque region, and the analysis is restricted to a specific sample of Lambayeque exporting SMEs. These factors may limit the generalisability of the findings to other regions of Peru or to different productive sectors. Furthermore, the utilisation of an econometric model founded upon binary logistic regression proffers a probabilistic approximation; nevertheless, it does not address other qualitative variables that could influence export competitiveness.

In terms of future lines of research, it is important to broaden the geographical scope of the study, to incorporate other dimensions of Business Intelligence, such as predictive analytics and Big Data, and to explore the impact of strategic alliances with technology companies on improving export competitiveness. Furthermore, the efficacy of market intelligence training programmes in enhancing the organisational capacities of Lambayequean SMEs and facilitating their internationalisation must be evaluated.

The promotion of innovation is considered a fundamental pillar for any company that aspires to consolidate a sustainable competitive advantage in international markets. In this sense, it is imperative that small and medium-sized enterprises (SMEs) direct their efforts towards investment in research and development (R&D), as well as in the adoption of technological advances. This, in effect, will not only allow them to optimise their export performance, but also facilitate the protection of their valuable information assets, a crucial aspect in today's dynamic global landscape.

Finally, the utilisation of external support constitutes a pivotal strategy for SMEs. By leveraging the various export promotion programmes at their disposal, and more crucially by cultivating robust relational norms, these companies can more adeptly circumvent the institutional barriers that frequently impede their progress. Indeed, this proactivity has been shown to engender significant improvements in coordination and responsiveness in the export arena, which is a key determinant of success in foreign markets.

## Ethical approval

This study was a minimal-risk, non-interventional investigation based on anonymous surveys in adults. It involved no clinical procedures, no experimental manipulation, and no collection of sensitive or directly identifiable data. The work was developed as voluntary academic research within the Graduate School of UNPRG and was not submitted as a thesis-/degree-related protocol for formal ethics committee review; therefore, no ethics committee/IRB approval, waiver, or reference number is available. Institutional ethics guidance is publicly available (Resolution No. 623-2021-CU and Resolution No. 624-2021-CU). The research adhered to the Declaration of Helsinki. Informed consent was obtained prior to participation (e-consent for the online form and written consent for in-person administration), and confidentiality and anonymity were protected through coding.

The study also adhered to UNPRG institutional ethics and scientific integrity guidance (including Resolution No. 1134-2018-R and the University Council Resolutions No. 623-2021-CU and No. 624-2021-CU).

## Data Availability

Zenodo. Business Intelligence and the export competitiveness of lambayecan SMEs in the north of Peru, https://doi.org/10.5281/zenodo.18517128. ^78^ This project contains the following underlying data:
• Database_ Information from data collected from SMEs (Data analysis was conducted using IBM SPSS Statistics 27 software). •
Data__BI_raw_results_presentation.xlsx. • Results of the research. https://zenodo.org/records/15621293/files/Results%20of%20the%20research.xlsx?download=1 Data is available under Creative Commons Zero v1.0 Universal This project contains the following extended data: Zenodo. Business Intelligence and the export competitiveness of lambayecan SMEs in the north of Peru, https://doi.org/10.5281/zenodo.18517128. ^78^
• Questionnaire (Yes/No) used for data collection. • Instrument file. • Informed consent • Sworn statement. •
STROBE_checklist_v4_combined. • Figures: ○
Figure 1. Global bibliometric metrics of scientific production in trade intelligence and exports according to Scopus (1996–2024). ○
Figure 2. Geographical location of Lambayeque (A: Peru; B: Lambayeque). ○
Figure 3. Sensitivity and specificity of the model. Data is available under Creative Commons Zero v1.0 Universal
