# Supplementary material for: Business Intelligence and the export competitiveness of lambayecan SMEs in the north of Peru
Source: F1000Res. 2026 Mar 6;15:368. [Version 1] doi: 10.12688/f1000research.167062.1 (PMC13122145; doi:10.12688/f1000research.167062.1)
Supplement: Peer review history

# Reviewer report: Reviewer response for version 1

**DOI:** 10.5256/f1000research.184139.r467916

**Published:** 2026-04-27

**Authors:** Ricardo Fernando Cosio Borda

**Affiliations:** 1 Universidad EAN, Bogotá, Colombia

The paper “Business Intelligence and the Export Competitiveness of Lambayecan SMEs in Northern Peru” presents a novel analysis from a general perspective, particularly in terms of its subject matter and target population.

 However, there are aspects that could be addressed to strengthen the paper, namely:

1. The Cronbach’s alpha coefficient for the instrument shows a low result that could be considered unacceptable. The authors state that 0.66 can be considered, in numerical terms, as 0.7, which would make it reliable. However, since the instrument is a fundamental element for collecting data that will lead to the results, it is not advisable to call it into question based on a result from the reliability assessment process. This is a point that is entirely within the researchers’ control.
2. It is appropriate to justify the use of a dichotomous scale for the Business Intelligence questionnaire. An instrument with a 5-point Likert scale would have been more appropriate.

Is the work clearly and accurately presented and does it cite the current literature?

Yes

If applicable, is the statistical analysis and its interpretation appropriate?

Partly

Are all the source data underlying the results available to ensure full reproducibility?

Partly

Is the study design appropriate and is the work technically sound?

Yes

Are the conclusions drawn adequately supported by the results?

Yes

Are sufficient details of methods and analysis provided to allow replication by others?

Partly

Reviewer Expertise:

Business, management, environment, international trade.

I confirm that I have read this submission and believe that I have an appropriate level of expertise to confirm that it is of an acceptable scientific standard, however I have significant reservations, as outlined above.

# Reviewer report: Reviewer response for version 1

**DOI:** 10.5256/f1000research.184139.r466078

**Published:** 2026-04-06

**Authors:** Marco Agustin Arbulu Ballesteros

**Affiliations:** 1 Ciencias empresariales., Universidad Cesar Vallejo (Ringgold ID: 488352), Trujillo, La Libertad, Peru

1. Small sample size and limited representativeness

 The study uses a sample of only 51 SMEs, drawn from a population of 106 exporting firms in Lambayeque. This number is insufficient to ensure the statistical stability of the binary logistic regression model, especially considering that the dependent variable exhibits a marked imbalance (76.5% “non-competitive” vs. 23.5% “competitive”). It is recommended to expand the sample or apply class imbalance correction techniques (such as SMOTE or weighting), and to more rigorously justify the calculation of the minimum required sample size.

 2. Insufficient operationalization of the main variables

 The three dimensions of Business Intelligence (business information, market research, and commercial management) are measured using dichotomous items (Yes/No), which considerably reduces the instrument’s discriminatory power. This simplification may partly explain why the “business information” dimension proved to be non-significant. It is recommended to replace or supplement the dichotomous scale with a Likert scale of at least 5 points, which would allow for capturing gradations in the actual use of BI tools, and to report the results of the confirmatory factor analysis that validates the proposed dimensional structure.

 3. Cronbach’s alpha coefficient below the acceptable threshold

 The authors report a Cronbach’s alpha of 0.66 and note that they consider it equivalent to 0.7, which is not methodologically correct: a value of 0.66 does not meet the standard reliability threshold (≥0.70). This inconsistency weakens the validity of the instrument. It is suggested that the questionnaire items be revised or refined to increase reliability, that alpha be reported by dimension (not just overall), and that complementary reliability metrics such as McDonald’s omega be considered, especially given the use of binary items.

 4. Absence of Causality Analysis and Control Variables

 The estimated Logit model includes only the three dimensions of BI as predictors, without incorporating relevant control variables such as firm size, years of export experience, the productive sector, or the manager’s educational level. Given the heterogeneity of the sample (9 distinct sectors, with a dominant agricultural sector accounting for 61%), these variables may act as confounders. It is recommended to include them as covariates in the model to better isolate the specific effect of BI on export competitiveness.

 5. Theoretical discussion disconnected from empirical findings

 The discussion section compares the results with the existing literature, but in several paragraphs the connection is superficial or forced. For example, Anjaningrum et al. are cited to point out a difference, but the study does not explore why Lambayeque SMEs differ structurally from the Indonesian firms analyzed in that study. Likewise, the conclusions derived from the model (odds ratios of 0.98 and 0.95) are interpreted optimistically when in reality they indicate a very modest marginal effect. It is recommended to deepen the contextual analysis of the divergences with the literature and to be more precise in interpreting the magnitude of the Logit model coefficients.

Is the work clearly and accurately presented and does it cite the current literature?

Yes

If applicable, is the statistical analysis and its interpretation appropriate?

Yes

Are all the source data underlying the results available to ensure full reproducibility?

Yes

Is the study design appropriate and is the work technically sound?

Yes

Are the conclusions drawn adequately supported by the results?

Yes

Are sufficient details of methods and analysis provided to allow replication by others?

Yes

Reviewer Expertise:

Ciencias empresariales.

I confirm that I have read this submission and believe that I have an appropriate level of expertise to confirm that it is of an acceptable scientific standard, however I have significant reservations, as outlined above.

# Reviewer report: Reviewer response for version 1

**DOI:** 10.5256/f1000research.184139.r466077

**Published:** 2026-03-24

**Authors:** Juan Alejandro Cortes

**Affiliations:** 1 Universidad Pontificia Bolivariana, Medellín, Colombia

Overall, this is a well-written paper, with particular strength in its methodological approach, especially in the quantitative domain. The literature review is robust and well aligned with the topic of the study.

 However, despite the overall quality of the manuscript, its focus on a specific region in northern Peru may limit its broader academic relevance unless cultural or social factors are explicitly analyzed and compared with findings from similar studies in other contexts.

 Additionally, the manuscript should be carefully reviewed, as there are a few sentences written in Spanish that need to be translated into English.

Is the work clearly and accurately presented and does it cite the current literature?

Yes

If applicable, is the statistical analysis and its interpretation appropriate?

Yes

Are all the source data underlying the results available to ensure full reproducibility?

Yes

Is the study design appropriate and is the work technically sound?

Yes

Are the conclusions drawn adequately supported by the results?

Yes

Are sufficient details of methods and analysis provided to allow replication by others?

Yes

Reviewer Expertise:

Organizational learningOrganizational designStrategic managementOrganizational cultureOrganizational structureManagement educationOrganizational changeBusiness strategy in Latin American contexts

I confirm that I have read this submission and believe that I have an appropriate level of expertise to confirm that it is of an acceptable scientific standard.

## References

[1] : The Concept of External Validity. *Journal of Consumer Research* .1982;9(3) : 10.1086/208920 10.1086/208920
